# Supplementary material for: The Possible Role of Anti- and Protumor-Infiltrating Lymphocytes in Pathologic Complete Response in Early Breast Cancer Patients Treated with Neoadjuvant Systemic Therapy
Source: Cancers (Basel). 2023 Sep 29;15(19):4794. doi: 10.3390/cancers15194794 (PMC10571934; doi:10.3390/cancers15194794)
Supplement: Supplementary file 1 [file cancers-15-04794-s001.zip › cancers-2590840-supplementary.pdf]

Supplementary Materials

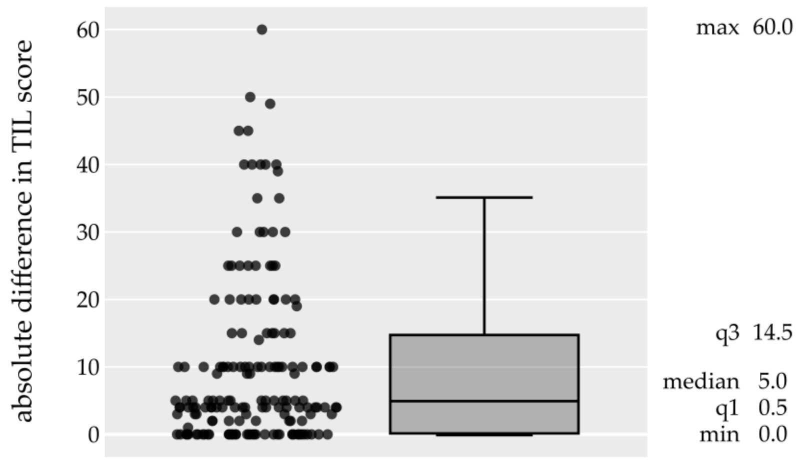

Figure S1: Absolute difference between TIL scores of two pathologists, boxplot.
Abbreviations: min: minimum; max: maximum; q3: 3rd quartile; q1: 1st quartile; TIL: tumor-infiltrating lymphocytes.

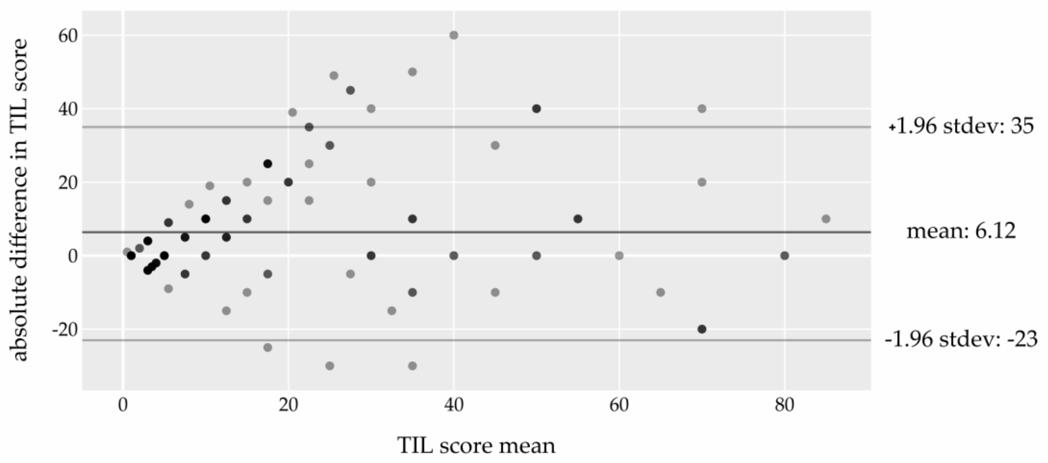

Figure S2: Bland-Altman plot for TIL measurements by two pathologists (solid line represents the mean difference, the dashed lines represent the 95% limits of agreement).
Abbreviations: TIL: tumor-infiltrating lymphocytes; stdev: standard deviation.

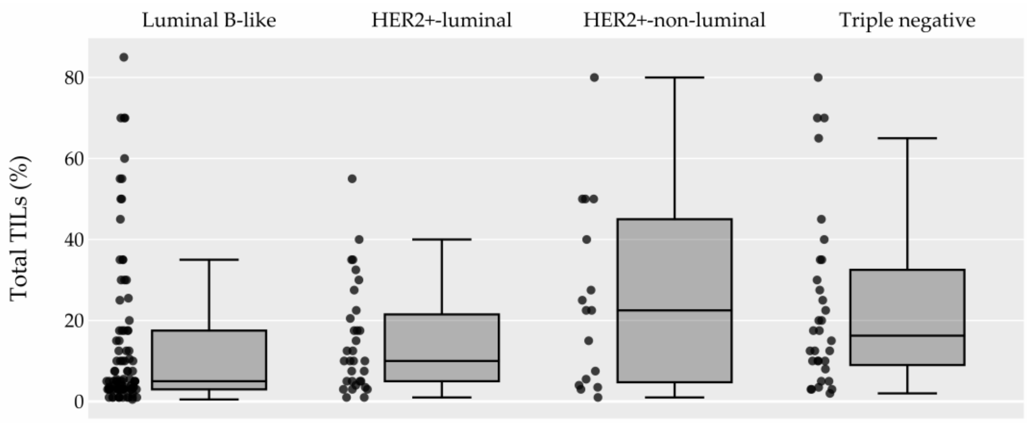

	Luminal B-like	HER2+-luminal	HER2+-non-luminal	Triple negative
max	85.0	55.0	80.0	80.0
q3	17.5	21.5	45.0	32.5
median	5.0	10.0	22.5	16.25
q1	3.0	5.0	4.75	9.0
min	0.5	1.0	1.0	2.0

Figure S3: Distribution of tumor-infiltrating lymphocytes in breast cancer according to the four breast cancer subtypes, boxplots. The difference in TIL between breast cancer subtypes on our sample (p=0.004) could not be generalized to the population (adjusted p=0.085), see Table S1.
Abbreviations: min: minimum; max: maximum; q3: 3rd quartile; q1: 1st quartile; TIL: tumor-infiltrating lymphocytes; HER-2: human epidermal growth factor receptor 2.

Table S1. Association of TIL and its subtypes (CD8+ TILs, CXCL13+ TILs, PD-1+ TILs, FOXP3+ TILs) with other clinicopathological characteristics.

Clinicopathologic characteristics	total TILs median (IQR)	p*	adjusted p-value †	total TILs n(%) low (<60%)	total TILs n(%) high (≥60%)	p #	adjusted p-value †	CD8+ TILs median (IQR)	p *	adjusted p-value †	CXCL13+ TILs median (IQR)	p *	adjusted p-value †	PD-1+ TILs median (IQR)	p *	adjusted p-value †	FOXP3+ TILs median (IQR)	p *	adjusted p-value †
**Grade 3**	12.5 (4.0-30.0)	<0.001	**0.002**	115 (93.0%)	9 (7.0%)	0.290	1.0	40.0 (30.0-50.0)	0.077	1.0	1.0 (0.0-2.0)	0.302	1.0	2.0 (0.0-7.0)	0.001	**0.015**	5.0 (2.0-7.5)	<0.001	**0.012**
**Grade 2**	5.0 (3.0-7.5)			45 (98.0%)	1 (2.0%)			30.0 (20.0-50.0)			1.0 (1.0-1.0)			0.0 (0.0-2.0)			2.0 (1.0-5.0)		
**Ki-67 10-20%**	5.0 (3.0-10.0)	<0.001	**0.002**	45 (98.0%)	1 (2.0%)	0.224	1.0	40.0 (30.0-50.0)	0.420	1.0	1.0 (0.5-2.0)	0.800	1.0	0.0 (0.0-2.0)	0.001	**0.024**	2.5 (1.0-5.0)	0.021	0.487
**Ki-67 20-40%**	10.0 (3.8-20.0)			63 (95.0%)	3 (5.0%)			40.0 (30.0-50.0)			1.0 (0.0-2.0)			1.0 (0.0-5.0)			4.0 (1.0-8.0)		
**Ki-67 >40%**	17.5 (5.5-35.0)			52 (90.0%)	6 (10.0%)			40.0 (27.5-50.0)			1.0 (1.0-2.0)			3.0 (2.0-8.0)			5.0 (2.0-7.0)		
**Lymph nodes positive**	10.0 (3.5-25.0)	0.284	1.0	117 (94.0%)	8 (6.0%)	1.000	1.0	40.0 (30.0-50.0)	0.259	1.0	1.0 (1.0-2.0)	0.546	1.0	2.0 (0.0-5.0)	0.740	1.0	5.0 (1.0-7.0)	0.840	1.0
**Lymph nodes negative**	6.5 (3.0-20.0)			44 (96.0%)	2 (4.0%)			40.0 (20.0-50.0)			1.0 (0.0-2.5)			1.5 (0.0-6.0)			3.0 (1.0-7.0)		
**Tumor ≤20 mm (MRI)**	30.0 (5.0-50.0)	0.051	1.0	22 (88.0%)	3 (12.0%)	0.305	1.0	40.0 (25.0-50.0)	0.887	1.0	1.0 (1.0-2.0)	0.002	0.051	3.0 (0.0-8.0)	0.609	1.0	5.0 (1.0-10.0)	0.709	1.0
**Tumor 20-50 mm (MRI)**	10.0 (3.0-22.5)			106 (94.0%)	7 (6.0%)			40.0 (30.0-50.0)			1.0 (0.0-2.0)			2.0 (0.0-5.0)			4.0 (1.5-7.0)		
**Tumor >50 mm (MRI)**	6.5 (4.0-11.2)			18 (100.0 %)	0 (0.0%)			40.0 (30.0-50.0)			2.0 (2.0-5.0)			1.0 (0.0-7.0)			5.0 (3.0-7.0)		
**Luminal B-like**	5.0 (3.0-17.5)	0.004	0.085	86 (95.0%)	5 (5.0%)	0.173	1.0	40.0 (30.0-50.0)	0.346	1.0	1.0 (1.0-2.0)	0.499	1.0	1.0 (0.0-5.0)	0.062	1.0	3.0 (1.0-6.5)	0.027	0.598
**HER2+ -luminal**	10.0 (5.0-21.5)			32 (100.0 %)	0 (0.0%)			45.0 (30.0-50.0)			1.0 (1.0-1.0)			1.0 (0.0-2.5)			3.0 (1.0-5.0)		
**HER2+ -non-luminal**	22.5 (4.8-45.0)			15 (94.0%)	1 (6.0%)			40.0 (30.0-60.0)			2.0 (0.0-3.0)			2.5 (0.0-8.5)			6.0 (3.5-9.0)		
**Triple negative**	16.2 (9.0-32.5)			28 (88.0%)	4 (12.0%)			40.0 (30.0-40.0)			1.0 (0.5-2.0)			3.0 (2.0-8.0)			6.0 (3.0-7.5)		

*Mann-Whitney test (if 2 categories) or Kruskal-Wallis test (if 3 categories or more); # Fisher's exact test; † p-values were adjusted for all comparisons in this table. *Statistically significant adjustet p-values are presented in bold.*

Abbreviations: TIL: tumor-infiltrating lymphocytes; HER-2: human epidermal growth factor receptor 2; IQR: interquartile range; n: number

Table S2: Multivariable logistic model of prognostic factors for pathological complete response (AUC= 0.807, loglikelihood ratio test p<0.001).

Characteristic	OR (95% CI)	p-value
**CD8+ TILs (per 10%)**	1.31 (1.01-1.72)	**0.045**
**Age**	0.98 (0.95-1.02)	0.351
**Ki-67 (per 10%)**	1.44 (1.18-1.79)	**<0.001**
**Negative lymph nodes**	2.34 (1.03-5.42)	**0.042**
**HER2+-luminal, HER2+-non-luminal, TN vs lumB**	4.93 (2.31-11.09)	**<0.001**

Statistically significant p-values are presented in bold.

TIL: tumor-infiltrating lymphocytes; TN: triple negative subtype; lumB: luminal B subtype; HER-2: human epidermal growth factor receptor 2; OR: odds ratio; CI: confidence interval; AUC: area under the curve

Table S3: Multivariable logistic model of prognostic factors for pathological complete response (AUC= 0.796, loglikelihood ratio test p<0.001).

Characteristic	OR (95% CI)	p-value
**CXCL13+ TILs (per 10%)**	1.01 (0.18-5.35)	0.934
**Age**	0.98 (0.95-1.01)	0.235
**Ki-67 (per 10%)**	1.39 (1.14-1.71)	**0.001**
**Negative lymph nodes**	1.93 (0.85-4.39)	0.114
**HER2+-luminal, HER2+-non-luminal, TN vs lumB**	4.79 (2.26-10.66)	**<0.001**

Statistically significant p-values are presented in bold.

TIL: tumor-infiltrating lymphocytes; TN: triple negative subtype; lumB: luminal B subtype; HER-2: human epidermal growth factor receptor 2; OR: odds ratio; CI: confidence interval; AUC: area under the curve

Table S4: Multivariable logistic model of prognostic factors for pathological complete response (AUC= 0.801, loglikelihood ratio test p<0.001).

Characteristic	OR (95% CI)	p-value
**PD-1+ TILs (per 10%)**	1.56 (0.87-2.83)	0.131
**Age**	0.98 (0.95-1.01)	0.262
**Ki-67 (per 10%)**	1.37 (1.12-1.69)	**0.002**
**Negative lymph nodes**	2.20 (0.97-5.08)	0.059
**HER2+-luminal, HER2+-non-luminal, TN vs lumB**	4.79 (2.26-10.67)	**<0.001**

Statistically significant p-values are presented in bold.

TIL: tumor-infiltrating lymphocytes; TN: triple negative subtype; lumB: luminal B subtype; HER-2: human epidermal growth factor receptor 2; OR: odds ratio; CI: confidence interval; AUC: area under the curve

Table S5: Multivariable logistic model of prognostic factors for pathological complete response (AUC= 0.807, loglikelihood ratio test p<0.001).

Characteristic	OR (95% CI)	p-value
**FOXP3+ TILs (per 10%)**	1.83 (0.73-4.61)	0.193
**Age**	0.98 (0.95-1.01)	0.213
**Ki-67 (per 10%)**	1.38 (1.13-1.70)	**0.001**
**Negative lymph nodes**	2.08 (0.92-4.72)	0.076
**HER2+-luminal, HER2+-non-luminal, TN vs lumB**	4.65 (2.21-10.27)	**<0.001**

Statistically significant p-values are presented in bold.

TIL: tumor-infiltrating lymphocytes; TN: triple negative subtype; lumB: luminal B subtype; HER-2: human epidermal growth factor receptor 2; OR: odds ratio; CI: confidence interval; AUC: area under the curve

Table S6: Multivariable logistic model of prognostic factors for pathological complete response (AUC= 0.811, loglikelihood ratio test p<0.001).

Characteristic	OR (95% CI)	p-value
**CD8+ TILs (per 10%)**	1.29 (0.96-1.76)	0.086
**CXCL13+ TILs (per 10%)**	0.55 (0.07-3.68)	0.533
**PD-1+ TILs (per 10%)**	1.31 (0.66-2.66)	0.429
**FOXP3+ TILs (per 10%)**	1.21 (0.39-3.63)	0.708
**Age**	0.98 (0.95-1.02)	0.328
**Ki-67 (per 10%)**	1.37 (1.12-1.71)	**0.002**
**Negative lymph nodes**	2.04 (0.88-4.78)	0.096
**HER2+-luminal, HER2+-non-luminal, TN vs lumB**	5.19 (2.40-11.92)	**<0.001**

Statistically significant p-values are presented in bold.

TIL: tumor-infiltrating lymphocytes; TN: triple negative subtype; lumB: luminal B subtype; HER-2: human epidermal growth factor receptor 2; OR: odds ratio; CI: confidence interval; AUC: area under the curve
